# Cardiotoxic effects of angiogenesis inhibitors

**DOI:** 10.1042/CS20200305

**Published:** 2021-01-06

**Authors:** Stephen J.H. Dobbin, Mark C. Petrie, Rachel C. Myles, Rhian M. Touyz, Ninian N. Lang

**Affiliations:** BHF Glasgow Cardiovascular Research Centre, Institute of Cardiovascular and Medical Sciences, University of Glasgow, 126 University Place, Glasgow, United Kingdom, G12 8TA

**Keywords:** atherosclerosis, cardiac arrhythmia, heart failure, hypertension, Tyrosine kinase inhibitors, vascular endothelial growth factor

## Abstract

The development of new therapies for cancer has led to dramatic improvements in survivorship. Angiogenesis inhibitors represent one such advancement, revolutionising treatment for a wide range of malignancies. However, these drugs are associated with cardiovascular toxicities which can impact optimal cancer treatment in the short-term and may lead to increased morbidity and mortality in the longer term. Vascular endothelial growth factor inhibitors (VEGFIs) are associated with hypertension, left ventricular systolic dysfunction (LVSD) and heart failure as well as arterial and venous thromboembolism, QTc interval prolongation and arrhythmia. The mechanisms behind the development of VEGFI-associated LVSD and heart failure likely involve the combination of a number of myocardial insults. These include direct myocardial effects, as well as secondary toxicity via coronary or peripheral vascular damage. Cardiac toxicity may result from the ‘on-target’ effects of VEGF inhibition or ‘off-target’ effects resulting from inhibition of other tyrosine kinases. Similar mechanisms may be involved in the development of VEGFI-associated right ventricular (RV) dysfunction. Some VEGFIs can be associated with QTc interval prolongation and an increased risk of ventricular and atrial arrhythmia. Further pre-clinical and clinical studies and trials are needed to better understand the impact of VEGFI on the cardiovascular system. Once mechanisms are elucidated, therapies can be investigated in clinical trials and surveillance strategies for identifying VEGFI-associated cardiovascular complications can be developed.

## Introduction

In recent years, survival for patients following a diagnosis of cancer has improved substantially. Almost 50% of patients diagnosed with cancer will now be alive 10 years later [1]. This remarkable improvement in cancer survivorship is, in large part, due to the increased anti-tumour effects of a rapidly broadening range of available cancer therapies. However, these advancements in treatment have been accompanied by numerous cardiovascular side effects which have led to an increase in morbidity and mortality influencing the long-term outlook for some patients. Because of the increasing use of newer anti-cancer drugs with the promise of prolonged survival, it is crucial that we advance knowledge on mechanisms underlying these toxicities to refine existing therapies, to define subpopulations of patients who might be at greater risk of cardiotoxicity and to guide potentially protective cardiovascular therapies. It remains essential that beneficial anti-cancer effects do not come at an unacceptable cardiovascular cost in the short-, medium- or long-term.

Angiogenesis inhibitors have revolutionised treatment for a wide range of malignancies. As tumours grow and become too large to be sufficiently perfused by the existing vasculature, hypoxic conditions stimulate tumour cells to produce proangiogenic cytokines including vascular endothelial growth factor (VEGF) [2]. Angiogenesis, the process of new blood vessel development, is essential for the growth and spread of solid cancer tumours and is regulated by promoting and inhibiting cytokines [3,4]. VEGF is fundamental for this process, making the VEGF signalling pathway an ideal therapeutic target for reducing cancer growth and metastases.

Following their rapid introduction, VEGF-signalling inhibitors (VEGFIs) have become increasingly associated with a range of cardiovascular toxicities. VEGFI-associated hypertension has received the most attention and a rise in blood pressure occurs in 80–90% of patients treated with VEGFI [5]. However, VEGFIs are also associated with a wider range of cardiovascular toxic effects including left ventricular systolic dysfunction (LVSD) and heart failure as well as arterial and venous thromboembolism, QT interval prolongation and arrhythmia [6,7]. The focus of this review will be upon putative mechanisms of VEGFI-associated cardiac toxicity, primarily LVSD and heart failure. However, we also outline other potential manifestations of VEGFI-associated cardiotoxicity including effects on coronary, peripheral and pulmonary vasculature, particularly where these may contribute to the development of VEGFI-associated LVSD. While major attention has been directed towards elucidating VEGFI effects upon cardiac contractile function, we also address the potential for cardiac toxicity to manifest as pro-arrhythmic effects. Before considering the potential cardiotoxic effects of VEGF inhibition in patients treated for cancer, we provide a primer on VEGF physiology and its role in cardiovascular physiology and disease.

## VEGF physiology and its role in cardiovascular diseases

**Figure 1:**
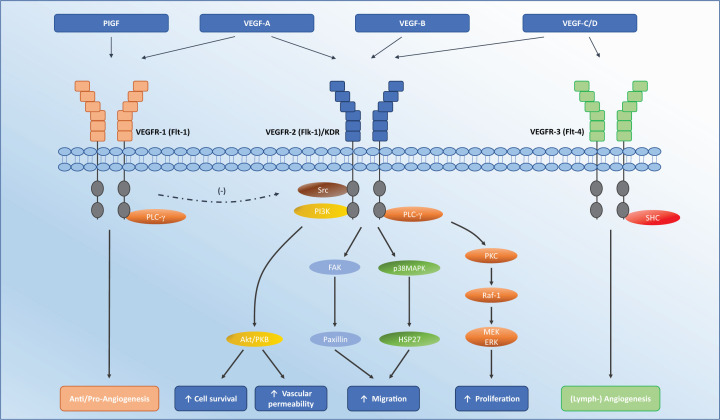
Interaction of VEGF ligands with their receptors VEGFR-1 may provide positive or negative feedback on angiogenesis depending on conditions; it inhibits embryonic angiogenesis and promotes pathological angiogenesis. It may also negatively regulate VEGFR-2 as per the (-) symbol. VEGF-A contributes to angiogenesis by activation of VEGFR2, leading to increased endothelial cell proliferation, migration and survival, and increased vascular permeability. Abbreviations: FAK, focal adhesion kinase; Flk-1, fetal liver kinase 1; Flt-1, Fms-like tyrosine kinase 1; HSP27, heat shock protein 27; KDR, kinase insert domain receptor; p38MAPK, p38 mitogen-activated protein kinase; PI3K, phosphatidyl inositol-3 kinase; PKC, protein kinase C; PLC-γ, phospholipase C; Raf, rapidly accelerated fibrosarcoma.

VEGF, a heparin-binding homodimeric glycoprotein, is produced by a variety of cell types including endothelial cells, fibroblasts, macrophages, platelets and tumour cells [8]. The VEGF family consists of VEGF-A, VEGF-B, VEGF-C, VEGF-D, and Placental Growth Factor (PlGF), of which VEGF-A is the best characterised and most associated with angiogenesis. There are three VEGF tyrosine kinase receptors (VEGFR-1, 2, and 3), of which, VEGFR-1 and -2 are predominantly expressed within the vasculature and activated by VEGF-A and VEGF-B, while VEGFR-3 is primarily expressed within the lymphatic system and activated by VEGF-C and VEGF-D (Figure 1) [9,10]. Binding of VEGF (mainly VEGF-A) to VEGFR-2 on endothelial cells triggers a series of phosphorylation cascades, which leads to downstream effects including increased vascular permeability, as well as cell migration, proliferation and survival (Figure 1) [11]. VEGF also plays an important role in the development, maintenance and survival of myocardial endothelial cells and cardiomyocytes [12,13]. This is particularly important in the context of cardiovascular disease, as myocardial stress and injury lead to increased VEGF secretion and up-regulation of VEGF signalling within cardiomyocytes [14,15].

### VEGF and the heart

#### Experimental models

The importance of VEGF in normal cardiac development and post-natal cardiac homoeostasis has been demonstrated in a variety of animal models [16]. VEGF enhances cardiomyocyte differentiation in mouse embryonic stem cells [17] and VEGF receptors are expressed on cultured neonatal rat cardiomyocytes [18]. Additionally, VEGF plays a role in cardiomyocyte turnover via activation of mitogen-activated protein kinase (MAPK) pathways, which play key regulatory roles in cell proliferation in rat cardiomyocytes [18]. VEGF may play a role in inducing post-natal cardiomyocytes to re-enter the cell cycle [18,19]. VEGF also serves as a compensatory mechanism in response to cardiac stressors and VEGF is secreted by cultured human cardiomyocytes in response to inflammation, ischaemia and hypertension [12,20,21]. Indeed, following aortic banding in rabbits, exogenous VEGF maintains myocardial angiogenesis and delays decompensation and decline in LV function [22]. When examined as a potentially anti-ischaemic therapy in pigs, VEGF increased the development of collateral coronary arteries and improved myocardial function [23–25].

Cardiomyocytes express VEGFR-1 and VEGFR-2, and both are up-regulated in response to hypoxia *in vitro* and *in vivo* [26]. The role of VEGF in myocardial remodelling is thought to be related to the balance of its actions on VEGFR-1, which inhibits the development of hypertrophy, and VEGFR-2, which has pro-hypertrophic effects [27,28]. A transgenic murine model of endothelial cell VEGFR-1 deletion led to angiogenesis and the development of cardiomyocyte hypertrophy via the tyrosine kinase, erb-B signalling [29]. These processes may be further influenced by microRNAs (miRNAs) such as miR-374, which positively regulate cardiomyocyte hypertrophy in mouse models [30].

#### Human studies

Following birth, human cardiomyocyte proliferation can be observed up to the age of 20 years [31,32]. However, throughout adulthood cardiac growth occurs via cardiomyocyte hypertrophy rather than hyperplasia. Myocardial hypertrophy may be physiological, in response to exercise or pregnancy, or pathological, as a maladaptive response to a chronic stimulus such as hypertension. Physiological hypertrophy is characterised by proportional capillary growth maintaining adequate capillary density, whereas pathological hypertrophy in adults is characterised by rarefaction of the coronary microvasculature [33]. Cardiac perfusion is largely controlled by the microvasculature through a dense capillary network with approximately one capillary per muscle fibre [34,35] and therefore, by virtue of its central role in neo-angiogenesis, VEGF plays a central role in cardiomyocyte hypertrophy. Post-mortem examination of hearts from patients with heart failure with preserved ejection fraction (HFpEF) reveals reduced microvascular density and increased myocardial fibrosis in comparison with control specimens from patients without heart failure [36].

In a study of 41 patients admitted with congestive heart failure, these patients had lower concentrations of circulating VEGF when compared with healthy controls [37]. Over the course of hospital admission for heart failure treatment, VEGF levels gradually returned to levels similar to control subjects without cardiovascular disease [37]. Additionally, endomyocardial biopsies from 21 patients with dilated cardiomyopathy reveal low levels of mRNA and protein expression of VEGF [38]. In contrast with patients with dilated (non-ischaemic) cardiomyopathy, biopsy specimens from 20 patients with ischaemia-related heart failure had higher levels of cardiomyocyte VEGF expression than healthy controls. Further highlighting the central importance of VEGF in myocardial perfusion, this higher myocardial VEGF expression was associated with a 58% increase in vascular density, while lower cardiac VEGF expression in patients with dilated cardiomyopathy was associated with a 66% decrease in vascular density when compared with healthy donor hearts. Following myocardial infarction (MI), patients who had improvement in LV function had higher levels of circulating VEGF than those with no improvement [39]. While a relative insufficiency of circulating VEGF in these conditions has prompted a series of clinical trials attempting to exploit the angiogenic properties of VEGF in patients with chronic coronary artery disease (CAD), these have, to date, been disappointing [40–42], although clinical investigation in this area continues [43].

### VEGF and the vasculature

#### Cell migration and proliferation

The potent mitogenic influence of VEGF on endothelial cells is mediated via VEGFR-2 and the activation of extracellular signal-regulated kinase (ERK1/2). Phosphorylation of VEGFR-2 leads to the activation of phospholipase C (PLC-γ), in turn stimulating the Raf-MAPK/ERK kinase (MEK)-ERK1/2 cascade in cultured endothelial cells [44,45]. Additionally, other MAPK pathways are activated by VEGF and are implicated in endothelial cell proliferation [46]. VEGF acts as a chemoattractant and plays a role in cell migration for a range of cultured cell types including vascular smooth muscle cells, monocytes and polymorphonuclear cells [11,47]. In cultured endothelial cells, cell migration is induced by activation of focal adhesion kinase (FAK) and paxillin, as well as via the phosphatidyl inositol kinase (PI3K)/Akt and MAPK pathways [48–50].

#### Cell survival

In addition to increasing endothelial cell proliferation and angiogenesis, VEGF plays a role in increasing endothelial cell survival and reducing apoptosis. Binding to VEGFR-2 activates the PI3k pathway and subsequent phosphorylation of Akt/protein kinase B via PDK1 in cultured human endothelial cells [51,52]. Akt phosphorylates Bcl2-associated death promoter and caspase 9, inhibiting their apoptotic activity and is therefore thought to mediate VEGF-related cell survival [53,54]. In cultured human endothelial cells, VEGF also induces the expression of other anti-apoptotic proteins including Bcl-2 and members of the inhibitors of apoptosis (IAP) family XIAP and survivin, which inhibit caspases 3 and 7 [55–57]. In addition to these ligand/surface receptor interactions, animal models demonstrate autocrine cell maintenance effects of VEGF within the endothelium [58,59].

#### Vascular permeability

VEGF was previously known as vascular permeability factor and its activation of VEGFR-2 on cultured endothelial cells triggers the activation of a series of pathways regulating the adhesive properties of the transmembrane protein, vascular endothelial-cadherin (VE-cadherin). These pathways include proto-oncogene tyrosine protein kinase src and culminate in the phosphorylation and internalisation of VE-cadherin, dissociating it from the cytoskeleton [60–63]. This consequent loss of the adherens junction results in a marked increase in vascular permeability [64] and these effects have been demonstrated in a variety of animal models with an increase in calcium-dependent hydraulic conductivity of microvessels [65,66], the opening of endothelial intercellular junctions and the induction of endothelial fenestration [67].

#### Vasodilatation

VEGF evokes potent endothelium-dependent vasodilatation, primarily via nitric oxide (NO) and prostacyclin (PGI_2_). In cultured human endothelial cells, binding of VEGF to VEGFR-2 activates the PI3K/Akt pathway with consequent endothelial NO synthase (eNOS) activation and NO release. NO production is up-regulated in human umbilical vein endothelial cells (HUVECs) after VEGF stimulation [68,69]. Additionally, activation of PLC-γ and subsequent activation of the Raf-MEK-ERK1/2 cascade activate eNOS as well as stimulating PGI_2_ release [70–72]. However, VEGF-induced vasodilatation is not abolished by NO and PGI_2_ inhibition in *ex vivo* human radial arteries, suggesting that endothelium-derived hyperpolarising factor (EDHF) is also implicated in VEGF-mediated vasodilatation [73].

## VEGF inhibition

As tumours grow, their core becomes hypoxic, stimulating the secretion of growth factors. VEGF is critically involved in this process and given its effects on the vasculature and, in particular, its role in neo-angiogenesis, inhibition of the VEGF pathways is a particularly attractive target for cancer therapy [74]. First introduced in 1971 following the ground-breaking work of Dr Judah Folkman [75], VEGFIs have become central to the treatment of many solid malignancies. Since the approval of the anti-VEGF monoclonal antibody, bevacizumab for use in colorectal cancer in 2004, the number of VEGFI agents and the malignancies they are used against has expanded substantially.

**Figure 2:**
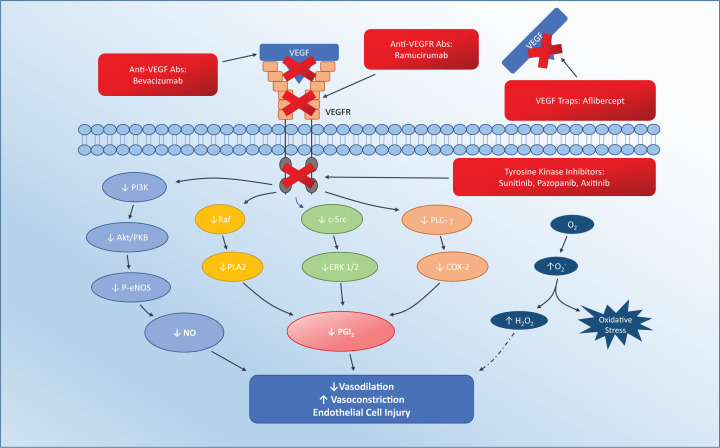
The pathogenesis of VEGF signalling inhibition-induced endothelial dysfunction via the different classes of VEGFI therapy and their site of action Disruption of PI3K/Akt/PKB and Raf-1/MEK/ERK pathways leads to reduced eNOS, NO and PGI2. This, combined with increased oxidative stress, leads to increased endothelial dysfunction. Abbreviations: cGMP, cyclic guanosine monophosphate, COX-2, cyclooxygenase-2, IP3, inositol-trisphosphate-3 kinase; P-eNOS, phospho-endothelial NO synthase; PI3K, phosphatidyl inositol-3 kinase; PLA2, phospholipase A2; Raf, rapidly accelerated fibrosarcoma.

Four classes of VEGFI have been developed: (1) monoclonal antibodies against VEGF; (2) monoclonal antibodies against VEGF receptors; (3) decoy VEGF receptors; (4) small molecule tyrosine kinase inhibitors (TKIs) (Figure 2 and Table 1). Numerous VEGF TKIs have been approved for use as first-line agents either as monotherapy or in combination with other anti-cancer therapies. Their use has been approved in a range of malignancies, including renal cell cancer (RCC), hepatocellular carcinoma (HCC), thyroid cancer, pancreatic neuroendocrine tumours, and soft tissue sarcoma (Table 1).

**Table 1:** Summary of VEGF inhibitors, their signalling targets and the cancers they are used to treat

Drug class	Agent	Target(s)	Clinical use
Monoclonal antibody	Bevacizumab	VEGF-A	Colorectal cancer
			Non-squamous non-small cell lung cancer
			Glioblastoma
			Renal cell carcinoma
	Ramucirumab	VEGFR-2	Colorectal cancer
			Gastric cancer
			Hepatocellular cancer
			Non-small cell lung cancer
Tyrosine kinase inhibitor	Axitinib	VEGFR-1, -2, -3	Renal cell carcinoma
		PDGFR	
		c-Kit	
	Cabozantinib	VEGFR-2	Medullary thyroid cancer
		RET	Renal cell carcinoma
	Cediranib	VEGFR-1, -2, -3	Colorectal cancer
		PDGFR	Glioblastoma
		c-Kit	Ovarian cancer
			Lung cancer
	Intedanib	VEGFR-1, -2, -3	Non-small cell lung cancer
		PDGFR	Ovarian cancer
		FGFR	
	Lenvatinib	VEGFR-1, -2, -3	Thyroid cancer
		PDGFR	Renal cell carcinoma
		c-Kit	
		RET	
		FGFR	
	Nintedanib	VEGFR-1, -2, -3	Non-small cell lung cancer
		PDGFR	
		RET	
		FGFR	
		FLT3	
	Pazopanib	VEGFR-1, -2, -3	Renal cell carcinoma
		PDGFR	Soft tissue sarcoma
		c-Kit	
	Regorafenib	VEGFR-1, -2, -3	Colorectal cancer
		PDGFR	
		c-Kit	
		RET	
		FGFR	
	Sorafenib	VEGFR-1, -2, -3	HCC
		PDGFR	Renal cell carcinoma
		Raf	Melanoma
		c-Kit	
		FLT3	
	Sunitinib	VEGFR-1, -2, -3	Gastrointestinal stromal tumour (GIST)
		PDGFR	Renal cell carcinoma
		c-Kit	Pancreatic neuroendocrine tumours (PNET)
		RET	
		FLT3	
	Tivozanib	VEGFR-1, -2, -3	Renal cell carcinoma
	Vandetanib	VEGFR-1, -2, -3	Medullary thyroid cancer
		PDGFR	Non-small cell lung cancer
		RET	
	Vatalanib	VEGFR-1, -2, -3	Colorectal cancer
		PDGFR	
		c-Kit	
VEGF-Trap	Aflibercept	VEGF-A/B	Colorectal cancer
		PIGF	
			

Abbreviations: FGFR, fibroblast growth factor receptor; FLT3, fms-like tyrosine kinase receptor 3; PDGFR, platelet-derived growth factor receptor; PlGF, placenta growth factor; Raf, rapidly accelerated fibrosarcoma; RET, rearranged during transfection; VEGFR, VEGF receptor.

### Clinical trials with VEGF TKIs

The oncological benefits of VEGF TKIs have been demonstrated in numerous clinical trials. For example, in a phase III trial of 750 patients with previously untreated metastatic RCC, treatment with sunitinib was associated with improved median progression-free survival in comparison with interferon-α (11 vs 5 months) [76]. In a trial of 1110 patients with advanced RCC treated with pazopanib or sunitinib, pazopanib was non-inferior to sunitinib and both are now recommended as first-line therapy for advanced RCC [77,78]. In addition to the use of VEGF TKIs as monotherapy, these drugs are increasingly used in combination with immunotherapy. A trial of 861 patients with metastatic RCC treated with the immunotherapy agent, pembrolizumab, in addition to axitinib or sunitinib demonstrated improved overall survival after 12 months follow-up (89.9 vs 78.3%) with pembrolizumab and axitinib compared with sunitinib [79]. However, longer term efficacy and safety data are awaited. Furthermore, VEGFI therapy is often used in patients who have received prior, potentially cardiotoxic, anti-cancer treatments such as anthracycline chemotherapy and radiotherapy. In a trial of 369 patients with progressive metastatic soft tissue sarcoma despite previous anti-cancer therapy, pazopanib improved progression-free survival when compared with placebo [80]. The approved VEGFI treatments and their indications are listed in Table 1.

### Targets of VEGF TKIs

VEGFIs exhibit both ‘on-target’ and ‘off-target’ effects determined by their degree of selectivity and mode of action. On-target effects are defined here as those resulting from the direct effects of VEGF pathway inhibition. Off-target effects are defined as those resulting from activity against other non-VEGF pathways. Monoclonal antibodies targeting circulating VEGF and its receptors and decoy receptors should be expected to be free from off-target effects because of their selective anti-VEGF mechanism (Figure 2). However, small molecule TKIs have activity against a much broader range of tyrosine kinase receptors in addition to the VEGF signalling pathway and thus have a much greater potential for ‘off-target’ effects (Table 1) [81]. Some of these off-target effects are intentional in order to broaden anti-tumour activity, but others may be unexpected and potentially unwanted. Taking sunitinib as an example, in addition to targeting VEGFR-1, -2, and -3, it has activity against platelet-derived growth factor receptors (PDGFR), and other receptor tyrosine kinases including c-kit, rearranged during transfection (RET), and fms-like tyrosine kinase-3 (FLT3). Defects in PDGF signalling are associated with tumour development and growth, and agents antagonising PDGF and its receptors have activity against a wide range of cancers [82,83]. C-kit receptors are expressed in many tumour types [84], and RET activation is also associated with malignancies such as thyroid cancers [85]. FLT3 activation is implicated in haematological malignancies, and FLT3 inhibitors have shown efficacy in treating these conditions [86,87]. Although these kinases are intended targets in tumour cells, inhibition of these signalling pathways in healthy cells, such as cardiomyocytes, may produce unintended toxicity.

## VEGF inhibition and LV dysfunction

### Definitions

The spectrum of the cardiotoxicity associated with VEGFI ranges from asymptomatic LVSD to heart failure, cardiogenic shock and death [6,88]. The true incidence of VEGFI-associated cardiotoxicity is poorly understood due to inconsistent definitions and reporting within clinical studies and clinical trials. Generally, a decrease in left ventricular ejection fraction (LVEF) below a pre-defined cut-off or an absolute drop in LVEF are most commonly used to define cardiotoxicity. However, the thresholds used for clinical decision-making vary between consensus guidelines [7,89–92]. Recent guidelines from the European Society of Medical Oncology (ESMO) provide a definition which has gained acceptance, albeit not universally. ESMO defines anti-cancer therapy-induced cardiotoxicity as a fall in LVEF of >10% from baseline to a value <50% [90]. This definition takes into consideration inter-observer and modality variability and the limitations of imaging modalities such as two-dimensional transthoracic echocardiography. Additionally, a drop in LVEF of >10% to an absolute LVEF of <50% may offer the most clinically relevant definition for patients undergoing cancer treatment although this has not been proven. In many of these patients, minor reductions in LVEF may be viewed as acceptable in the context of potentially life-prolonging anti-cancer therapy. A reduction in LVEF beyond this threshold may require intervention to avoid adverse cardiovascular outcomes and to allow the continued safe delivery of anti-cancer therapy. LV dysfunction associated with VEGFIs typically occurs early in treatment and the introduction of cardioprotective therapies (usually angiotensin-converting enzyme [ACE] inhibition and β blockade) even for mildly reduced LV function can restore LVEF [93,94]. This, therefore, allows for potential continuation or re-challenge of VEGFI therapy.

### Incidence of VEGFI-associated cardiotoxicity

A meta-analysis of 3784 patients with breast cancer treated with bevacizumab in randomised, controlled trials reported an incidence of ‘high grade’ congestive heart failure of 1.6% with bevacizumab and 0.4% with placebo. The term ‘high-grade’ refers to grade 3 or higher heart failure as per the National Cancer Institute (NCI) common terminology criteria for adverse events, which includes patients with LVEF < 40% and a spectrum of clinical presentations from patients symptomatic on minimal exertion to cardiogenic shock and death [92]. Although the incidence was low, there was a relative risk (RR) of developing symptomatic heart failure of 4.74 with the addition of bevacizumab to anti-cancer regimens when compared with the addition of placebo [95] This provides evidence for the ‘on target’ cardiotoxic effect of VEGF inhibition, given that this monoclonal antibody against VEGF would not be expected to have off-target effects.

Meta-analysis of trials including a total of 10647 patients treated with TKIs (including axitinib, pazopanib, sorafenib, sunitinib and vandetanib) in the context of a range of malignancies reveals a combined incidence of asymptomatic LVSD of 2.4%, with 1.2% developing symptomatic heart failure. Of the 21 trials included in the meta-analysis, only 5 had baseline and protocol-mandated follow-up cardiac imaging (Table 2) [6]. Many trials relied on a clinical definition of heart failure, with no quantification of LVSD, and no incidence of asymptomatic LV impairment. Furthermore, the incidence of LVSD and HF was appreciably higher in trials with protocol-mandated follow-up imaging than those without, highlighting the probable under-reporting of LVSD. This is particularly relevant given the high degree of selection bias in these trials, of which almost all excluded patients with a history of cardiovascular disease. Of note, there was no difference in the risk of cardiotoxicity between more specific TKIs (e.g. axitinib) and those with activity against a broader range of tyrosine kinases (e.g. sunitinib, sorafenib, vandetanib and pazopanib) [6]. However, these were not direct head-to-head comparisons between these TKIs. A larger meta-analysis including 28538 patients treated with targeted cancer therapies, including all four classes of VEGFIs, provided similar results. TKIs were associated with the highest RR for ‘high-grade’ cardiotoxicity at 5.62 when compared with cancer patients receiving placebo or alternative anti-cancer treatment. Notably, ramucirumab (a monoclonal antibody directed against VEGFR-2) and aflibercept (a decoy VEGF receptor) were also among the agents with the highest relative risk of ‘high-grade’ cardiotoxicity at 5.0 and 4.1 respectively when compared with the same controls [96]. Although the RR of cardiotoxicity was high with monoclonal antibodies and may be counterintuitive given the anticipated lower or absent incidence of off-target effects, these comparisons were also not head-to-head with TKIs, and there was heterogeneity between treatment regimens. Therefore, it is not possible to draw a definitive conclusion from these data.

**Table 2:** Randomised trials of VEGF TKI therapy, the cardiac monitoring performed during them and the incidence of congestive heart failure [6]

Author, year	Histology	Number of patients enrolled	Treatment arms	Age (range), years	Pre-existing cardiovascular disease included	Cardiac function monitoring	Definition of LVSD/HF	Number of all-grade CHF	Number of grade ≥3 CHF	Events reported
Abou-alfa, 2010	HCC	96	Doxorubicin + sorafenib	66 (38–82)	No	Baseline MUGA/echo; Follow-up criteria not specified	CTCAE v3.0	9 (19%)	1 (2%)	LV dysfunction
			Doxorubicin + placebo	65 (38– 81)				1 (2%)	0	
Barrios, 2010	HER2-negative breast cancer	478	Sunitinib	53 (25–80)	No	Baseline MUGA/echo; Follow-up at regular intervals	CTCAE v3.0	5 (2.1%)	NR	Cardiac failure, congestive cardiac failure
			Capecitabine	53 (23– 80)				0	NR	
Bergh, 2012	HER2-negative breast cancer	593	Docetaxel + sunitinib	54 (31– 84)	No	Baseline MUGA/echo; Follow-up as clinically indicated and at discontinuation	CTCAE v3.0	3 (1.3%)	3 (1.3%)	Cardiac failure
			Docetaxel	56 (28– 78)				0	0	
Boer, 2011	NSCLC	534	Pemetrexed + vandetanib	60 (28– 82)	No	Not specified	CTCAE v3.0	1 (0.4%)	NR	Acute cardiac failure
			Pemetrexed + placebo	60 (35– 83)				0	NR	
Carrato, 2013	Colorectal cancer	768	FOLFIRI + sunitinib	59 (25– 83)	No	Baseline MUGA/echo; Follow-up as clinically indicated	CTCAE v3.0	1 (0.3%)	NR	Cardiac failure, congestive cardiac failure
			FOLFIRI + placebo	58 (25– 82)				3 (0.8%)	0	
Crown, 2013	Breast cancer	442	Capecitabine + sunitinib	52 (27– 79)	No	Baseline MUGA/echo; Follow-up at each cycle	CTCAE v3.0	1 (0.5%)	NR	Acute cardiac failure, congestive cardiomyopathy
			Capecitabine	54 (31– 77)				1 (0.5%)	NR	
Demetri, 2006	GIST	361	Sunitinib	58 (23– 84)	Not specified	Baseline MUGA; Follow-up at each cycle	CTCAE v3.0	22 (10%)	2 (1%)	Decreased ejection fraction, LV dysfunction
			Placebo	55 (23– 81)				2 (2%)	0	
Escudier, 2007	RCC	903	Sorafenib	58 (19– 86)	No	Not specified	CTCAE v3.0	1 (0.2%)	NR	LV dysfunction
			Placebo	59 (29– 84)				0	0	
Flaherty, 2013	Melanoma	823	Cardoplatin + paclitaxel + sorafenib	60 (19– 86)	No	Not specified	CTCAE v3.0	1 (0.3%)	NR	LV dysfunction
			Carboplatin + paclitaxel + placebo	59 (18– 84)				2 (0.5%)	NR	
Gridelli, 2012	NSCLC	124	Gemcitabine + vandetanib	75 (NR)	No	Not specified	CTCAE v3.0	1 (1.64%)	NR	Acute cardiac failure
			Gemcitabine + placebo	75 (NR)				0	0	
Haas, 2016	RCC	1943	Adjuvant sunitinib	56 (49– 64)	No	Baseline MUGA; Follow-up at 3, 6, 12 months and as indicated	CTCAE v3.0	5 (1%)	5 (1%)	LV dysfunction
			Adjuvant sorafenib	55 (48– 63)				5 (1%)	5 (1%)	
			Placebo	57 (49– 64)				2 (0.3%)	2 (0.3%)	
Kindler, 2011	Pancreatic cancer	630	Gemcitabine + axitinib	61 (34– 84)	No	Not specified	CTCAE v3.0	0	0	Cardiac failure
			Gemcitabine + placebo	62 (35– 89)				2 (0.6%)	2 (0.6%)	
Lencioni, 2012	HCC	307	TACE + Doxorubicin-eluting beads + sorafenib	64.5 (NR)	Not specified	Not specified	CTCAE v3.0	1 (0.7%)	NR	LV dysfunction
			TACE + Doxorubicin-eluting beads + placebo	63 (NR)				2 (1.3%)	NR	
Llovet, 2008	HCC	602	Sorafenib	64.9 (NR)	No	Not specified	CTCAE v3.0	1 (0.3%)	NR	LV dysfunction
			Placebo	66.3 (NR)				2 (0.7%)	NR	
Michaelson, 2014	Castration-resistant Prostate cancer	873	Prednisone + sunitinib	69 (39– 90)	No	Baseline MUGA/echo; Follow-up as clinically indicated	CTCAE v3.0	6 (0.5%)	NR	Cardiac failure, congestive cardiac failure, LV dysfunction
			Predisone + placebo	68 (47– 86)				0	0	
Motzer, 2013	RCC	1110	Sunitinib	62 (23– 86)	No	MUGA/echo at baseline; Follow-up every 3 cycles	CTCAE v3.0	42 (11%)	4 (1%)	LV dysfunction, heart failure
			Pazopanib	61 (18– 88)				47 (13%)	4 (1%)	
Motzer, 2009	RCC	735	Sunitinib	62 (27– 87)	No	Baseline MUGA; Follow-up at regular intervals	CTCAE v3.0	61 (16%)	10 (3%)	Decreased ejection fraction
			Interferon	59 (34– 85)				19 (5%)	6 (2%)	
Paz-Ares, 2012	NSCLC	904	Gemcitabine + cisplatin + sorafenib	60 (28– 81)	No	Not specified	CTCAE v3.0	2 (0.4%)	NR	LV dysfunction
			Gemcitabine + cisplatin + placebo	58 (22– 77)				1 (0.2%)	NR	
Raymond, 2011	PNET	171	Sunitinib	56 (25– 84)	No	Baseline MUGA/echo; Follow-up as clinically indicated	CTCAE v3.0	2 (2%)	1 (1%)	Cardiac failure
			Placebo	57 (26– 78)				0	0	
Rugo, 2011	Breast cancer	168	Docetaxel + axitinib	55 (30– 79)	No	Not specified	CTCAE v3.0	2 (1.8%)	1 (0.9%)	Ventricular hypokinesia
			Docetaxel + placebo	56 (34– 71)				0	0	
Sternberg, 2010	RCC	435	Pazopanib	59 (28– 85)	No	Not specified	CTCAE v3.0	1 (0.3%)	NR	Cardiac failure
			Placebo	60 (25– 81)				0	0	
Van der Graaf, 2012	Soft-tissue sarcoma	369	Pazopanib	56.7 (20.1– 83.7)	No	Baseline MUGA/echo; Follow-up after 12 weeks then every 8 weeks	CTCAE v3.0	16 (11%)	NR	Decrease in ventricular function
			Placebo	51.9 (18.8– 78.6)				2 (5%)	NR	
Wells, 2012	Medullary thyroid cancer	331	Vandetanib	50.7 (NR)	No	Not specified	CTCAE v3.0	1 (0.4%)	NR	Acute cardiac failure
			Placebo	53.4 (NR)				0	0	

In a randomised controlled trial of 1110 patients comparing pazopanib with sunitinib for the treatment of RCC, the incidence of heart failure was 1% in both treatment groups, while 9% of patients in each group had a ≥15% decline in LVEF [77]. In the ASSURE trial (adjuvant sunitinib or sorafenib vs placebo in resected unfavourable renal cell carcinoma), 1599 patients with RCC were treated with sunitinib, sorafenib or placebo. There was a reduction in LVEF > 15% to below the lower limit of normal in 1.8 and 1.4% for sunitinib and sorafenib respectively, and in 0.9% receiving placebo over 6 months [97].

Overall, it is likely that the incidence VEGFI-associated LVSD and heart failure has historically been underestimated. Many analyses have been based on clinical trial datasets, which are often highly selective and exclude patients with pre-existing cardiovascular disease. Additionally, cardiovascular risk factors have often been poorly reported in clinical trials. Higher rates of baseline comorbidity may be expected within the ‘non-trial’ or ‘real world’ cancer population and may predispose to higher rates of cardiotoxicity. The true incidence of VEGFI-associated LVSD may therefore range from 10 to 20% with the incidence of heart failure being approximately 1–5%. Retrospective analysis of 159 real-world patients treated with targeted therapies including VEGFIs, at Stanford University found 14% had a decline in LVEF of ≥10% from baseline during treatment [98]. The majority of these changes were asymptomatic, with only 3% developing symptomatic heart failure. These patients had a substantial prevalence of baseline comorbidity, which is likely to explain the higher incidence of cardiotoxicity than is reported in clinical trials. However, these registry data were also limited by incomplete imaging follow-up (only 56% had any LVEF assessment), despite a local monitoring algorithm recommending regular follow-up imaging. Therefore, the incidence of cardiotoxicity is likely to have been underestimated even in this population.

### Time course of VEGFI-associated cardiotoxicity in patients

Cardiotoxicity associated with VEGFIs is at least partially reversible. In one study, 90 patients treated with sunitinib for RCC underwent prospective echocardiographic and biomarker assessment. A total of 9.7% of patients developed a reduction in LVEF by ≥10% to a value <50% when compared with baseline. Almost all of those who developed LVSD did so within the first cycle of treatment. However, with sunitinib dose reduction and/or treatment with cardiac medicines (variably including ACE inhibitor, β blocker and/or dihydropyridine calcium channel blocker), LVSD was at least partially reversible up to 33 weeks follow-up [99]. Such reversibility in VEGFI-associated LVSD was also evident in a randomised controlled trial of sunitinib against placebo for imatinib-resistant gastrointestinal stromal cancer. In this trial, 28% of patients had a decline in LVEF of ≥10% with 8% developing congestive heart failure and there was a steady decline over the 24-week follow-up. However, all patients who developed congestive heart failure achieved an improvement in LVEF and resolution of symptoms with dose adjustment and heart failure therapies [100]. Two of those who developed heart failure underwent endomyocardial biopsy, which demonstrated cardiomyocyte hypertrophy and abnormal mitochondrial configurations, but no evidence of apoptosis or fibrosis [100]. Given the potentially reversible nature of VEGFI-associated LVSD, this supports the role of routine prospective monitoring of patients treated with VEGFIs. Early detection of LVSD may increase the likelihood of reversibility and, in turn, may allow for the reintroduction of VEGFI treatment, potentially at a lower dose and in the presence of conventional therapies used for the treatment of heart failure, including renin–angiotensin–aldosterone system inhibitors and β-blockers. Further evidence is required to determine the optimum timing, frequency and benefits of such a monitoring strategy.

## Mechanisms of VEGFI cardiotoxicity

**Figure 3:**
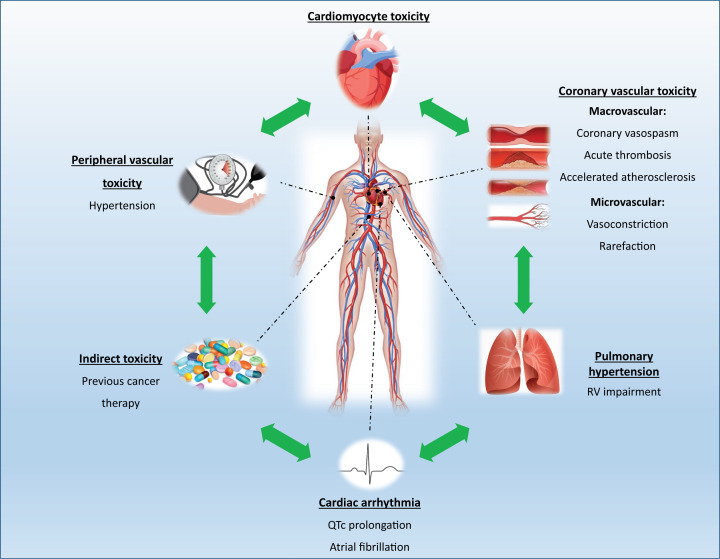
Mechanisms of VEGF inhibitor-induced cardiotoxicity Abbreviation: RV, right ventricular.

The pathophysiological mechanisms underlying VEGFI-induced cardiotoxicity remain poorly defined but, in view of the central role of VEGF in cardiovascular physiology, it is not surprising that the on-target consequences of VEGF inhibition invoke unwanted cardiac effects. Off-target effects may be of particular relevance in the development of cardiotoxicity associated with VEGF TKIs. Additionally, indirect effects secondary to previous or concurrent exposure to other cardiotoxic agents such as anthracyclines or the development of VEGFI-associated systemic hypertension may accelerate the development of LV dysfunction (Figure 3) [101].

### Cardiomyocyte toxicity

#### On-target effects

VEGF plays a key role in myocardial contractility in animal models. Binding of VEGF and subsequent activation of PLC-γ controls myocardial contractility by regulating calcium cycling within cultured rat ventricular cardiomyocytes [102]. Additionally, the recessive lethal zebrafish mutation, *dead beat (ded)*, on the *zplcγ1* gene causes myocardial dysfunction and a loss of coronary vasculature. Zebrafish embryos are not dependent on a circulatory system early in development because passive diffusion provides sufficient oxygen delivery [103]. As a result, cardiac-specific expression of wildtype *zplcγ1* in these *ded* mutants was shown to restore myocardial function despite a lack of coronary vasculature [102]. This suggests that, in addition to its role in vasculogenesis, VEGF has a direct role in cardiomyocyte contractility. These findings are supported by mouse models only expressing the VEGFA_120_ gene. Of these mice, only a proportion survive till birth and, those that do survive have left ventricular dilatation and impaired contractility [104]. Similarly, in mice with cardiomyocyte-specific VEGF knockout, *in utero* survival is limited and those that do survive to birth have ventricular wall thinning and impaired cardiac function [105]. Collectively, these pre-clinical data provide convincing evidence to suggest that VEGFI could be expected to have direct cardiomyocyte toxic effects. Whether this effect, in isolation, is enough to cause clinically evident LVSD in humans is unknown but more likely to represent one important component of a multifaceted mechanism.

#### Off-target effects

**Figure 4:**
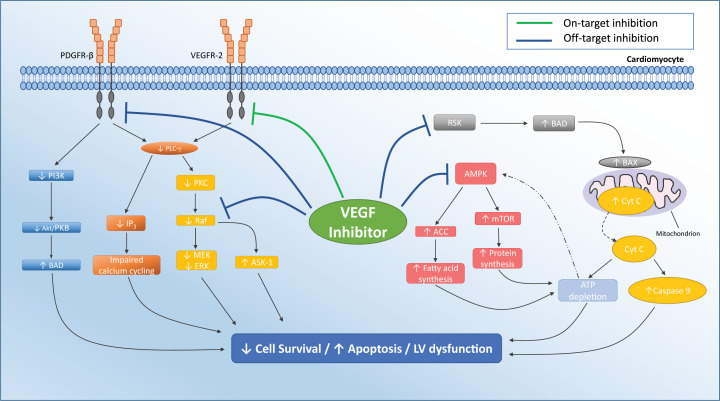
Pathways by which VEGF inhibitors cause direct myocardial toxicity Inhibition of ribosomal S6 kinase (RSK) may lead to activation of the pro-apoptotic factor BAD. This leads to BAX activation and Cyt C release from mitochondria. This, in turn, leads to ATP depletion and activation of caspase 9 and can lead to apoptosis. Normally, ATP depletion would lead to activation of AMP kinase (AMPK); however, VEGFI inhibition of AMPK prevents this action, leading to increased activation of ACC and mTOR activity and further ATP depletion by fatty acid and protein synthesis. Inhibition of PDGFR-β leads to decreased PI3K, which may lead to up-regulation of pro-apoptotic BAD pathway. Direct inhibition of Raf-1 may down-regulate the MEK-ERK pathway, reducing cell survival. Additionally, Raf-1 inhibition may prevent the inhibition of pro-apoptotic kinases such as ASK-1. On-target inhibition of VEGFR-2 may down-regulate PLC-γ leading to reduced IP3 and impaired calcium cycling, which may reduce myocardial contractility. Abbreviations: ACC, acetyl-coenzyme A carboxylase; ASK-1, apoptosis-signal-regulating kinase-1; BAD, BCL2-antagonist of cell death; BAX, BCL2-associated X protein; Cyt C, cytochrome *c*; IP3, inositol-trisphosphate-3 kinase; mTOR, mammalian target of rapamycin; PI3K, phosphatidyl inositol-3 kinase; PLC- γ, phospholipase C; PKC, Protein kinase C; PDGFR-β, platelet-derived growth factor receptor; Raf-1, rapidly accelerated fibrosarcoma-1.

Other direct myocardial toxic effects of VEGFIs, particularly VEGF TKIs, may be attributed to off-target effects upon pathways other than those associated with binding of VEGF to its receptors (Figure 4). It has been estimated that VEGF TKIs dysregulate approximately 80 kinases in the hearts of mouse models [106]. It is therefore likely that there are other kinases targeted by VEGFIs which play a role in the development of cardiotoxicity yet to be discovered.

#### Adenosine monophosphate kinase

Adenosine monophosphate kinase (AMPK) down-regulation may be an off-target effect of VEGFI. AMPK is critical to cardiomyocyte energy homoeostasis. AMPK is activated when ATP levels decrease and inhibits energy consuming pathways, including protein and fatty acid synthesis (Figure 4). AMPK also activates energy-generating pathways via fatty acid oxidation and glycolysis and maintains mitochondrial homoeostasis, through mitophagy and biogenesis in cultured cardiomyocytes [107,108]. VEGF TKIs inhibit AMPK in cultured rat cardiomyocytes, depleting cardiomyocyte energy stores [109]. In cultured rat cardiomyocytes, treatment with sunitinib is associated with the release of cytochrome *c* from mitochondria and activation of caspase-9, both pivotal in the initiation of the mitochondrial apoptotic pathway [100]. In addition to these downstream effects on mitochondrial function, sorafenib also causes direct mitochondrial dysfunction within cultured rat cardiomyocytes [110].

In mice treated with sunitinib, trimetazidine reverses cardiotoxicity and improves cardiomyocyte viability via activation of the AMPK/mTOR/ribosome S6 kinase/autophagy pathway [111–113]. Trimetazidine selectively inhibits mitochondrial long chain 3-ketoacyl-CoA thiolase, which in turn inhibits β-oxidation of fatty acids, promoting glucose utilisation and improving cardiac energy metabolism [114]. This more efficient energy turnover makes the myocardium more durable to conditions of energy shortage as seen with the effects of VEGFIs on AMPK activity.

Cardiomyocytes of mice treated with sunitinib exhibit mitochondrial swelling and degenerative changes such as whorls and loss of cristae [100]. Endomyocardial biopsy specimens from three patients with VEGFI-associated cardiotoxicity revealed similar mitochondrial abnormalities, but without apoptosis or fibrosis [100,109]. Repeat biopsy after discontinuation of treatment and initiation of heart failure therapies showed a marked improvement in mitochondrial and overall cardiac function [109].

#### Platelet-derived growth factor receptor

The non-specific effects of VEGF TKIs include inhibition of PDGFR. Pazopanib is a potent inhibitor of PDGFR-β in cultured tumour cells [115], and sunitinib inhibits PDGFR-β phosphorylation in mouse tumour models [116,117]. PDGFR-β is expressed on the cell surface of mouse cardiomyocytes and endothelial cells and plays a role in angiogenesis [118–120]. PDGFR-β is also up-regulated in mouse models of left ventricular pressure overload via transverse aortic constriction (TAC). Although PDGFR-β has not been considered to be required for normal cardiac development or function, in mouse models, cardiac-specific PDGFR-β knockout is associated with worse left ventricular function and less angiogenesis after TAC in comparison with wild-type mice after TAC [118]. Furthermore, following induction of MI in rat models, delivery of PDGF was shown to improve cardiac function [121]. This exposure of the potential importance of PDGFR in the context of TAC has parallels with the clinical context in which direct myocardial toxic effects might otherwise be inconsequential but are instead potentiated and unmasked by concurrent systemic hypertension.

#### Rapidly accelerated fibrosarcoma-1

Rapidly accelerated fibrosarcoma-1 (Raf-1) is expressed throughout embryonic development and functions via activation of the MEK-ERK1/2 pathway. Raf-1 activation of this pathway is associated with cell proliferation and inhibition of apoptosis *in vitro* [122,123]. It also has actions outside this kinase pathway and inhibits pro-apoptotic kinases such as apoptosis signal-regulating kinase 1 (ASK-1) and mammalian Ste20-like kinase (MST-2) [122]. In addition to its actions on VEGFRs, sorafenib is a potent inhibitor of Raf-1. This was initially demonstrated *in vitro*, where sorafenib inhibited Raf-1 in tumour cells and subsequently has been shown in human xenografts of breast, colon and lung cancer implanted in mouse models [124,125]. In cultured neonatal rat ventricular cardiomyocytes and zebrafish, Raf-1 inhibition with sorafenib is associated with myocardial apoptosis and impaired cardiac function. In this model, apoptosis is attenuated by up-regulation of MEK-ERK activity suggesting a role for Raf-1-MEK-ERK signalling in cardiotoxicity [126].

Raf-1 is not essential for normal murine cardiac development [127,128]. However, cardiac-specific Raf-1 knockout is associated with LV dilatation and myocardial dysfunction in mice by 5 weeks of age as well as cardiomyocyte apoptosis and fibrosis [128]. These changes can be prevented by ASK-1 knockout suggesting RAF-1 inhibition of ASK-1 may regulate myocardial function [128]. Another mouse model of cardiac-specific expression of the dominant-negative form of Raf-1 was not associated with abnormal resting cardiac function [113,127]. However, similar to the situation encountered with PDGFR knockout mice, when exposed to pressure overload via TAC, there was substantial myocardial apoptosis and mortality as a result of LV dysfunction [127]. Therefore, the kinase activity of Raf-1 may be important in the response of cardiomyocytes to stressors, while its interactions with pro-apoptotic signals may help maintain cardiomyocyte survival and function.

#### c-kit

VEGF TKIs target the stem cell growth factor (SCF) receptor, c-kit which, although not expressed on cardiomyocytes, is involved in the myocardial response to injury [129]. Sunitinib inhibits c-kit in both tumour cell culture and mouse tumour models and this inhibition is associated with reduced tumour cell proliferation and survival [116,117]. C-kit is expressed on endothelial progenitor cells as well as cardiac and haematopoietic stem cells and is important for the migration of these cells to sites of injury [130]. *In vitro* studies of human cardiac progenitor cells demonstrate that c-kit signalling promotes cell growth, survival and proliferation [131]. Additionally, in mutant mouse models of c-kit dysfunction there is impaired cardiac recovery following MI and decline in cardiac function with ageing [129,132–134]. Given these findings, it has been postulated that inhibition of its activity may upset the normal cardiac response to injury within human myocardium. However, a variant of imatinib which does not target Abl, but retains activity against c-kit, was not associated with cardiac dysfunction in a mouse model. This was attributed to the lack of Abl inhibition [135] and casts some doubt on the role of c-kit inhibition in the development of VEGFI-induced cardiotoxicity.

### Coronary vascular toxicity

The incidence of myocardial ischaemia in patients treated with VEGFIs is relatively low. In a meta-analysis of 4617 patients, those treated with bevacizumab had a significantly increased risk of ischaemic heart disease (defined as MI, unstable angina, coronary revascularisation, coronary artery disease, arrhythmias, sudden death or cardiovascular death) compared with controls treated with concurrent chemotherapy or placebo (RR: 2.49). However, the overall incidence of ischaemic heart disease was only 1.0% [136]. These effects are not limited to bevacizumab though. Another larger meta-analysis examined the cardiovascular events of patients treated with a wide range of VEGFIs in 72 studies (*n*=38078). Of the trials where fatal and non-fatal MI were reported (*n*=4163), VEGFIs had a significantly increased risk of MI (RR: 3.54) when compared with controls, although the absolute risk was only 0.8% [137]. Similar results were observed in a subsequent meta-analysis of VEGFIs, which reported an incidence of cardiac ischaemia of 1.7% in 3891 patients treated with VEGFIs (RR: 2.83). However, the parameters for defining cardiac ischaemia were not described, and although 77 studies were included in the analysis, only 8 recorded cardiac ischaemic events sufficiently to allow for comparison [138]. This, therefore, limits the robustness of these data and highlights the inconsistency of reporting of cardiovascular events within these clinical trials.

#### Coronary macrovascular effects

VEGFIs have been associated with a range of coronary arterial effects including accelerated atherosclerosis, vasospasm and acute thrombosis.

#### Accelerated atherosclerosis

The role of VEGF, and its inhibition, in the development and progression of atherosclerosis has been debated. In the setting of VEGF inhibition, human endothelial cells *in vitro* have increased mitochondrial superoxide generation and reduced NO production, which may predispose to atherosclerosis [139]. VEGF inhibition in apolipoprotein E knockout mice receiving a high cholesterol diet accelerates atherosclerosis progression but does not alter plaque vulnerability [139]. However, other *in vivo* studies of rabbit models of atherosclerotic plaque induction have reported the inhibition of plaque neovascularisation and progression in those treated with bevacizumab, particularly in the early stages of plaque formation [140,141]. Consistent with this, plaque progression and destabilisation has been reported in mice and rabbits treated with recombinant VEGF [142]. There have however been case reports of progression of CAD and plaque rupture with VEGFI therapy [143,144]. The opposing effects noted in different studies and settings may be explained by variations in local VEGF concentrations. While low levels of VEGF may be required for vascular homoeostasis, higher levels may stimulate vascular proliferation. As such, inhibition of physiological levels of VEGF may accelerate atherosclerosis, whereas inhibition of supraphysiological levels may provide therapeutic benefit.

Pre-existing CAD has been identified as a potential risk factor for the development of VEGFI-induced cardiotoxicity [100,145]. This may be related to the concept of perfusion–contraction mismatch, which suggests that myocardial contraction is related to myocardial blood flow [146,147]. Changes in myocardial perfusion may not always result from a focal coronary stenosis restricting blood flow. The combination of diffuse CAD throughout epicardial coronary arteries, insufficient to warrant intervention when taken individually, can lead to reductions in myocardial blood flow and perfusion pressure [148]. VEGFIs may play a role in the development or worsening of this mismatch by accelerating the process of atherosclerosis through the suppression of NO production.

The presence of macrovascular CAD is a marker for coexisting microvascular disease, processes associated with oxidative stress and inflammation [149,150]. Microvascular dysfunction can also contribute to the increase in vascular resistance with CAD further compromising myocardial blood flow [148,151]. Therefore, the presence of CAD in major epicardial coronary arteries is more likely to act as a risk factor for VEGFI-induced LV dysfunction because it represents a more generalised myocardial vulnerability rather than an elevated risk because of macrovascular coronary stenoses *per se*. While plaque rupture or epicardial vasospasm might result in acute MI and LVSD, this acute effect could be considered as a separate, albeit related pathogenic process in VEGFI-associated LVSD.

#### Coronary vasospasm

As outlined, in normal circumstances VEGF provokes endothelial release of NO through its action on VEGFR-2 and this is interrupted by VEGFI. Administration of anti-VEGFR2 antibody in mice caused reduced expression of eNOS when compared with wild ype mice [152]. Functional assessment of the effects of sunitinib in rats demonstrated a reduction in endothelium-dependent vasorelaxation, and similar changes were found in animals treated with an NOS inhibitor [153]. An alternative suggested mechanism involves coronary smooth muscle Rho kinase, which is up-regulated by sorafenib in rat models [154]. In pigs, Rho kinase plays an important role in the development of vasospasm through augmentation of calcium sensitisation in coronary smooth muscle cells [155].

In humans, VEGFI treatment of 31 patients with gastrointestinal stromal tumours suppresses the bioavailability of NO and this recovers within 1 week of withdrawal of treatment [156]. The endothelial dysfunction resulting from these changes is a potential substrate for susceptibility to coronary vasospasm [157]. Indeed, VEGFI-induced coronary vasospasm, including consequent MI has been reported in patients receiving VEGFI therapy [158–160].

#### Acute thrombosis

The pro-coagulant state of cancer may be worsened by VEGFIs, which increase the risk of arterial thrombosis and venous thromboembolism further [138,161]. Haemostasis is regulated by a balance of coagulant, fibrinolytic and platelet-activating and inhibiting factors. VEGFI-induced suppression of endothelial NO and PGI_2_ reduces their anti-platelet effects [162]. Additionally, VEGF inhibition increases the haematocrit in experimental models as a result of increased erythrocytosis from production of erythropoietin in hepatocytes [163]. This increased viscosity may also increase the risk of thromboembolic events. Furthermore, in mouse models, bevacizumab forms immune complexes with VEGF, which activate platelets via FcγRIIa receptors in a manner similar to heparin-induced thrombocytopaenia [164]. Heparin administration increases the deposition of bevacizumab–VEGF immune complexes on platelets and thus platelet activation. Furthermore, the use of thrombin in mice to simulate hypercoagulable states, such as in cancer, reduces the amount of heparin required to trigger platelet activation [164].

#### Coronary microvascular effects

Coronary perfusion is largely controlled by the microvasculature, and alterations in its structure and function have been linked to a range of cardiac diseases [165]. VEGFIs influence large vessels and the microvasculature. Indeed, their effects upon the microvasculature may be of greater overall importance than those on larger vessels with a closer interaction with VEGFI-associated cardiomyocyte toxicity and vulnerability to relative ischaemia. Microvascular changes may produce a chronically progressive cardiotoxic process while large arterial issues may be more abrupt, for example in the setting of arterial thrombosis or spasm and MI. Reductions in the myocardial capillary network can lead to cardiac hypoperfusion, which may contribute to myocyte death and fibrosis [166]. Mice with cardiac-specific VEGF knockout have fewer coronary microvessels [105], reduced angiogenesis and capillaries that are tortuous and dilated [104]. In rats treated with sunitinib, coronary flow is reduced and responsiveness of the coronary vasculature to sodium nitroprusside is attenuated within 8 days of treatment [167]. Mice treated with sunitinib have similar coronary microvascular dysfunction and associated loss of pericytes. Similar effects were observed in mice treated with PDGFR inhibitors, suggesting PDGFR inhibition by VEGF TKIs may play a role in the development of microvascular dysfunction [118].

The molecular mechanisms by which these microvascular changes induce LVSD, as well as a deep understanding of the interaction between microvascular dysfunction and direct VEGFI-associated cardiomyocyte toxicity, remain to be fully defined. However, activation of hypoxia inducible factor-α (HIF-α) may be a central component. HIF-α accumulates in murine cardiomyocytes during hypoxic conditions and, via dimerisation with HIF-β, acts as a transcription factor to activate genes for cellular adaptation to hypoxia [168]. In the presence of oxygen, HIF-α is inhibited by a prolyl hydroxylase domain (PHD), which induces hydroxylation and degradation. In rodent models, in the acute setting, activation of HIF-α reduces myocardial injury in ischaemia [169–171], but chronic HIF-α activation induces dilated cardiomyopathy [168,172]. VEGFI-induced microvascular dysfunction may induce a chronic hypoxic state and activation of HIF-α and this may be a particularly potent cause of LVSD in the setting of other VEGFI-associated mechanisms of cardiomyocyte dysfunction. Indeed, a transgenic mouse model of myocardium-specific tetracycline-induced soluble VEGF decoy receptor (VEGFR-1) reversibly induces myocardial ischaemia without infarction via functional loss of capillaries. In this model, hypoperfusion resulted in HIF-α activation, mitochondrial autophagy and LVSD, which was completely reversible with removal of the VEGF trap [173].

The process of rarefaction, the loss of capillaries, has been observed in patients treated with VEGFIs. Whether this process is structural, indicating a permanent loss, or a potentially reversible functional one is unclear. In a study of 14 patients with breast and colorectal cancer treated with bevacizumab, rarefaction of sublingual capillaries was observed after 6 weeks of bevacizumab treatment. However, these changes were reversible on repeat imaging 3 months after bevacizumab was discontinued [174]. The duration of treatment was not reported, and whether, over time, VEGFI treatment eventually leads to a structural loss of capillaries from an initially functional one remains unclear. Similarly, our own prospective study of ten patients receiving VEGF TKI therapy showed reduced myocardial blood flow at rest on stress-perfusion cardiac magnetic resonance imaging (CMR) following 4 weeks of treatment [175]. These changes were corrected by adenosine-induced myocardial stress, suggesting this process may be a result of functional vasoconstriction rather than a structural loss. In 18 patients treated with sunitinib, echocardiographic measures of coronary flow reserve were lower than in healthy controls [176]. In the absence of epicardial CAD, these changes reflect microvascular dysfunction and, consistent with this, microvascular angina has been reported in patients treated with bevacizumab [177]. These changes in perfusion, coupled with chronic activation of HIF-α may play a role in the development of VEGFI cardiotoxicity.

### Peripheral vascular effects and indirect cardiac toxicity

The most clearly defined cardiovascular effect of VEGFI is hypertension. While the evidence above outlines the potential for direct effects of VEGFI on the myocardium and coronary vasculature, it would be expected that these cardiotoxic effects are potentiated substantially by the increased, and potentially sudden, rise in blood pressure following VEGFI treatment. Indeed, it is highly probable VEGFI-induced cardiotoxicity is a ‘multiple hit’ phenomenon as a result of direct cardiomyocyte toxicity, coronary micro- and macro-vascular disease, potentiated by increased left ventricular afterload due to systemic hypertension (Figure 3). Furthermore, it is likely that previous or concurrent treatment with potentially cardiotoxic anti-cancer therapies such as anthracyclines creates a ‘vulnerable myocardium’ predisposing to LV dysfunction when exposed to subsequent VEGFI therapy [178].

#### Hypertension

Hypertension is the commonest cardiovascular toxicity associated with VEGFIs. New, or worsening of pre-existing hypertension, has been reported in up to 80% of patients treated with VEGFIs [5]. From registry data, 73% of patients receiving targeted therapy (predominantly VEGF TKIs) for RCC developed cardiovascular toxicity, 55% of which was accounted for by hypertension [98]. This can, in some cases, be severe and difficult to treat, impacting on the dose and duration of therapy. The mechanisms of VEGFI-induced hypertension have been extensively reviewed previously [179–181], and we provide a brief overview of these and how they may contribute to VEGFI-associated cardiotoxicity. Given the rapid onset (within days of commencing treatment), and the reversibility on cessation, it is thought that changes in vascular tone play an important role [156].

Reduced NO bioavailability is a potentially important factor in VEGFI-associated hypertension. In mice, VEGFR-2 inhibition reduced eNOS expression and NO activity leading to hypertension [152]. Additionally, VEGFI treatment in swine is associated with a rise in the potent vasoconstrictor, endothelin-1 (ET-1) in association with a rise in blood pressure. Notably, these effects were reversed by ET-1 antagonism [182]. More recent pre-clinical studies have shown that VEGF inhibition may also increase reactive oxygen species (ROS) production through NADPH oxidase (Nox) dysregulation and down-regulation of the antioxidants within endothelial cells [183]. Acetylcholine-mediated vasodilatation was reduced in mice treated with vatalanib, and these effects were ameliorated by the ROS scavenger N-acetyl-cysteine (NAC) [183]. However, the significance of these findings in the clinical setting require further elucidation. Although the renin–angiotensin–aldosterone (RAA) system is key in the pathophysiology of hypertension, there is no convincing evidence it plays a major role in VEGFI-associated hypertension [184]. The changes in capillary density within the myocardium have also been shown in the systemic circulation. Whether this is structural, due to a loss of capillaries from local thrombosis, or a functional process as a result of increased vasomotor tone, remains unclear. However, these microvascular changes have been linked to increasing vascular resistance, and therefore contributing to the development of hypertension [185]. Mice treated with VEGFI have a demonstrable reduction in tracheal capillary density within 24 h of initiation of VEGF inhibition, which progresses over time [186].

In humans, similar changes in peripheral capillary density have been noted with VEGFIs, and persisted beyond 6 months in patients during treatment with bevacizumab [187–189]. These changes were associated with a rise in blood pressure. However, importantly, there is recovery of capillary density following discontinuation of treatment, suggesting a more functional process [174,189]. In patients with gastrointestinal stromal tumours, regorafenib therapy is associated with suppression of NO and increase in ET-1 [156]. Additionally, functional studies assessing endothelial-dependent vasodilatation in patients treated with telatinib and bevacizumab found that this was impaired and was also associated with an increase in blood pressure [188,189]. The extent of the role vasoconstriction and rarefaction play in the development of VEGFI-induced hypertension remains unclear and whether cardiac rarefaction occurs in the same way and with the same temporal dynamics are not completely defined.

#### Other cancer therapies and the risk of VEGFI-associated cardiotoxicity

##### Anthracyclines

While many other classes of anti-cancer therapies are associated with potential cardiotoxic effects, the toxic effects of anthracyclines are most prominent. Doxorubicin stimulates VEGF release from cardiac microvascular endothelial cells and ventricular cardiomyocytes [190] and this may confer some protection from anthracycline-related cardiotoxicity. Indeed, cultured cardiomyocytes engineered to overexpress VEGF-A_165_ were protected from doxorubicin-induced apoptosis via an increase in Bcl-2 expression and up-regulation of the Akt–NF-B pro-survival pathway [191]. Additionally, overexpression of VEGF-B in mice treated with doxorubicin protects cardiomyocytes as well as the cardiac microvasculature [192]. Furthermore, while the evidence base is not substantial, prior exposure to this chemotherapy class has been associated with an increased risk for VEGFI-associated cardiotoxicity [193]. It is unclear whether VEGFI therapy in the context of prior or concurrent anthracycline exposure simply results in additive risk or whether any shared mechanisms of cardiotoxicity amplify these unwanted actions.

Bevacizumab has been linked to an increased risk for LVSD and heart failure when combined with anthracycline therapy. In the MAIN trial (phase III, randomised, placebo-controlled, rituximab plus bevacizumab in aggressive Non-Hodgkin's Lymphoma) 787 patients were treated with R-CHOP (rituximab, cyclophosphamide, doxorubicin, vincristine, and prednisolone) in addition to bevacizumab or placebo [178]. These patients underwent echocardiography at 4-month intervals for 12 months, with those developing LVSD or heart failure followed-up every 3 months until resolution. This revealed that those treated with R-CHOP plus bevacizumab had a three-fold increased risk of LVSD and heart failure compared with those treated with R-CHOP plus placebo with an incidence of 18 vs 8% and 16 vs 7%, respectively [178]. Furthermore, a study of 210 patients treated with anthracyclines and bevacizumab concurrently reported an incidence of ‘high-grade’ congestive heart failure of 2.8% and a RR of 6.22 compared with placebo [95]. However, details of cardiac imaging follow-up were not reported, and the incidence of LVSD may therefore have been higher than reported.

Therefore, these data lend support to the hypothesis that VEGFI could potentiate anthracycline-induced cardiotoxicity by inhibition of protective VEGF release. This hypothesis remains to be fully explored and whether this is of relevance when exposure to VEGFI is delayed after anthracycline therapy is not clear.

##### Immune checkpoint inhibitors

Immunotherapy for cancer treatment has brought enormous cancer survival benefits and evidence for the use of immune checkpoint inhibitors (ICIs) is growing rapidly. ICIs have been associated with myocarditis and this has become a major focus for research. While its incidence is apparently relatively low, with reports from trial databases and registries ranging from 0.09 to 2% [194,195], up to half of cases are fatal [196]. ICIs are now increasingly used in combination with VEGFIs. In a phase Ib study of 55 patients with RCC treated with a PD-L1 inhibitor (avelumab) and axitinib, there was a 2% incidence of fatal myocarditis [197]. In a phase III trial comparing combination of a PD-1 inhibitor (pembrolizumab) and axitinib with sunitinib in 861 patients with metastatic renal cell cancer 2 cases (0.5%) of myocarditis were reported, one of which was fatal in the pembrolizumab and axitinib group (0.2%) [79]. There were also two cases of cardiac failure (0.5%), however there was no protocol-mandated cardiac imaging performed and the incidence of LV dysfunction may have been higher [79].

Although there has not been clear overlap in the mechanisms underlying ICI-associated myocarditis and VEGFI-associated cardiotoxicity, the potential impact of one upon the other is becoming increasingly relevant. Given the cardiotoxic potential of both these agents, it is possible that LV dysfunction caused by one, may be amplified by the other. Furthermore, with the potentially enormous survival gains achieved by some patients with immunotherapy, the cardiovascular effects of much more prolonged VEGFI therapy will become clearer.

### Limitations of pre-clinical mechanistic models

Common risk factors for heart failure and cancer such as pre-existing hypertension, smoking, diabetes, and obesity make the myocardium more vulnerable to the cardiotoxic effects of anti-cancer therapy [198]. These issues limit some of the observations from pre-clinical models attempting to define mechanisms of VEGFI-associated cardiotoxicity as they cannot fully replicate the multi-morbidity and complexity of the host environment of a patient treated for cancer. Additionally, most pre-clinical studies of VEGF therapies focus on anti-tumour effects without systematic examination of coexisting cardiovascular effects and this may be a potentially wasted opportunity. Furthermore, inter-species differences also pose limitations. For example, monoclonal antibodies such as bevacizumab have no activity against rodent VEGF [199].

**Figure 5:**
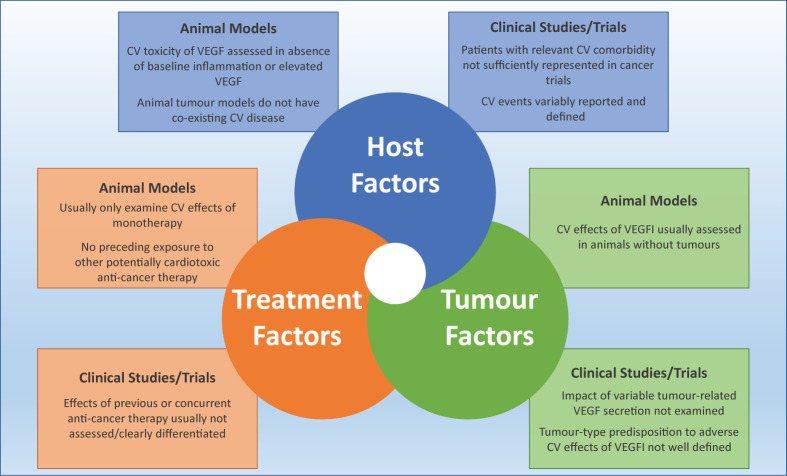
Limitations posed by pre-clinical and clinical models of efficacy and safety of VEGFI therapy Abbreviation: CV, cardiovascular.

Pre-clinical studies examining potential toxicities are usually performed in the absence of cancer, and therefore do not replicate the potentially important interactions between VEGFI therapy and tumour-related VEGF secretions, inflammation or oxidative stress. Some tumours evoke much greater VEGF secretion [200,201] and baseline levels of VEGF may substantially affect the cardiotoxic, as well as anti-cancer, potency of these drugs. This is exemplified by the correlation between substantially better cancer outcomes in patients developing a hypertensive response to VEGFI treatment when compared with those who do not have a rise in blood pressure [202]. It remains unknown whether such a correlation between anti-tumour effects, circulating VEGF concentration and the development of LVSD exists. The potential development of VEGFI-associated cardiotoxicity is likely to reflect the complex interplay between host factors (baseline and incident comorbidity and possible genetic factors), tumour-specific factors (including paracrine and distant hormonal effects [particularly VEGF secretion]), as well as histological type, site and stage (Figure 5).

## VEGF inhibition and the right heart

VEGF is abundant within the pulmonary vasculature where it is important for homoeostasis [203]. Up-regulation of VEGF and its receptors in the lungs can be seen in animal models of pulmonary hypertension (PH) as well as in patients [204–206]. However, whether VEGF plays a causal role in the development of PH, or is a compensatory mechanism is unclear. Overexpression of VEGF in rat models does not induce PH, and blunts the development of hypoxia-induced PH. Additionally, PH in a rat model of pulmonary fibrosis was ameliorated by VEGF, whereas it was exacerbated by VEGF inhibition [207].

In addition to direct effects on the pulmonary vasculature, VEGF inhibition may contribute to the development of PH via indirect mechanisms. VEGFIs are associated with an increase in pulmonary venous thromboembolism [137,208], and this risk is additional to the risk posed by the hypercoagulable state associated with cancer and patients are at risk from chronic thromboembolic pulmonary hypertension (CTEPH). Animal models suggest a role for VEGF in resolution of thromboemboli [209,210] and thrombus resolution is delayed in mice with endothelial cell-specific deletion of VEGFR-2 [211].

VEGF is up-regulated in pre-clinical models of PH and right ventricular (RV) hypertrophy [212–214]. In animals with RV failure, VEGF levels are decreased and associated with myocardial capillary rarefaction [212,215]. Furthermore, exposure to VEGFI is associated with the development of RV systolic failure in chronically hypoxic rats with RV pressure overload [212]. Severe PH and right heart failure has been reported following 18-month-treatment with bevacizumab in a patient with previously normal pulmonary artery pressures and right heart function [216]. There was no evidence of lung disease or pulmonary thromboembolism and the underlying mechanisms remain unexplained. In a study assessing the RV myocardium of 41 patients, samples were obtained from patients with normal RV function, compensated RV hypertrophy and decompensated RV hypertrophy. Patients were categorised on the basis of clinical history and tricuspid annular plane systolic excursion (TAPSE) on echocardiography. Samples from patients with normal RV function were obtained following surgery for aortic stenosis or at autopsy following coronary artery bypass grafting or sudden death due to CAD. Patients with compensated RV hypertrophy all had a history of congenital heart disease and normal LV function with samples obtained at the time of cardiac surgery. Those with decompensated RV hypertrophy had PH with RV failure and samples were obtained at autopsy. Biopsies from patients in all three groups had similar levels of VEGF and VEGFR-2. However, those with decompensated RV failure had capillary rarefaction when compared with patients with normal RV function and compensated RV hypertrophy. This was attributed to up-regulated sprout-related EVH1 domain-containing protein-1 (SPRED-1), an inhibitor of phosphorylated-ERK 1/2, which is a downstream product of VEGFR-2 activation [217]. This suggests VEGF inhibition may impair the ability of cardiomyocytes within the RV to respond to myocardial stress, precipitating RV dysfunction. Therefore, similar to the processes thought to be responsible for VEGFI-associated LV dysfunction, the development of RV failure with VEGFIs is likely to be related to a ‘multiple-hit’ mechanism with effects on cardiomyocytes, the coronary, pulmonary and venous vasculature.

## VEGF inhibition and arrhythmia

VEGFIs have been associated with a variable incidence of QT-interval prolongation, bradycardia and tachycardias. Tachyarrhythmias include monomorphic and polymorphic ventricular tachycardia (VT) and there is emerging evidence of VEGFI-associated atrial fibrillation (AF). The true incidence of arrhythmia with VEGFIs is likely to be underestimated given cardiac monitoring is not routinely performed with therapy.

Sinus bradycardia has been reported in up to 19% of patients treated with pazopanib within clinical trials [218]. When used in combination with vorinostat, a histone deacetylase inhibitor, bevacizumab was associated with sinus bradycardia in 3% of patients [219]. There have also been case reports of high-grade atrioventricular (AV) block in patients treated with VEGFIs requiring implantation of a permanent pacemaker [220,221], but their true incidence and pathogenic link to VEGFI are undefined.

### QTc prolongation and Torsades de Pointes

Drug-induced QTc prolongation is associated with a risk of Torsades de Pointes (TdP), a specific form of polymorphic VT which causes rapid haemodynamic compromise and may degenerate to ventricular fibrillation (VF). TdP causes syncope and carries a high risk of arrhythmic sudden cardiac death (SCD). The relationship between QTc and risk of TdP is complex and influenced by a series of dynamic variables but, in general, a QTc > 500 ms is accepted to be associated with a clinically significant risk of ventricular arrhythmia and SCD.

Different VEGFI agents have been observed to have varying effects on QTc intervals. VEGF TKIs most associated with QTc prolongation include sorafenib, sunitinib and vandetanib. Conversely, there are no reports within clinical trials of QTc prolongation with bevacizumab, and a trial of 87 cancer patients treated with aflibercept, which has a higher affinity for binding VEGF, demonstrated a mean increase in QTc of only 8.4 ms [222]. This suggests the effects on QTc interval are unrelated to the inhibition of VEGF signalling. In a meta-analysis of 6548 patients, the incidence of all-grade and high-grade QTc prolongation (>500 ms and/or TdP) was 4.4 and 0.83% respectively in patients treated with VEGF TKIs [223]. Many studies, however, did not perform routine electrocardiogram (ECG) monitoring, and the incidence of QTc prolongation may be higher, particularly as vandetanib trials, which had the highest incidence of QTc prolongation, also had the highest frequency of ECG monitoring. There was also heterogeneity between the studies with respect to whether patients with baseline QTc prolongation and those taking other QTc-prolonging drugs were included, which further complicates interpretation of the results for clinical practice. Another pooled analysis of 280 patients treated with sorafenib or sunitinib reported an average incidence of all-grade QTc prolongation and QTc > 500 ms of 8.5 and 1.9% respectively [224]. Additionally a meta-analysis of patients treated with vandetanib found an incidence of all-grade and high-grade (QTc > 500 ms) QTc prolongation of 16.4 and 3.7% respectively in patients with non-thyroid cancer, and 18 and 12% respectively in those with thyroid cancer [225], underlining that even the same drug may have markedly different effects on the QTc interval when used for differing indications, with the inherent differences in treatment duration, drug combinations and underlying pathophysiology. The extent to which key QTc modulators such as heart rate, age, gender and electrolyte status could be factored into risk assessments and treatment protocols is also unclear.

Although QTc prolongation is linked to an increased risk of ventricular arrhythmias such as TdP, and there have been reports of ventricular arrhythmia with VEGFI therapy, the incidence is rare. The reported incidence of ventricular arrhythmia or TdP in one meta-analysis was 0.1% [223]. This risk needs to be balanced against the valuable anti-cancer effects of this class of drugs.

Most drug-induced QTc-prolongation is thought to be caused by direct blockade of the pore-forming subunit of the cardiac potassium channel which carries the rapid delayed rectifier current (IKr), which is the major determinant of repolarisation in ventricular myocytes. A reduction in IKr retards repolarisation, leading to prolongation of the ventricular action potential (AP), which is manifest as QTc prolongation on ECG. QTc interval prolonging effects of VEGFI were first noted during pre-clinical studies [226]. As with most other drugs VEGFIs are thought to cause QTc prolongation via direct inhibitory effects on the human ether-a-go-go related gene (hERG) channel. For example, pre-clinical studies reveal that vandetanib causes hERG block at low concentrations and is associated with both AP prolongation in cardiac tissue preparation and QTc prolongation *in vivo* suggesting a classical drug–channel interaction [226]. However, given the vast array of tyrosine kinases dysregulated by these agents, it is likely that there are complex mechanisms by which they contribute to QTc prolongation and these are the focus of ongoing studies. Future advances in our mechanistic understanding of coupled with more detailed clinical data should inform the safe and judicious use of these agents.

### Atrial Fibrillation

AF has most commonly been reported with the VEGF TKI sorafenib. The incidence of AF was 5.1% when used in combination with 5-fluorouracil in a study of 39 patients with advanced HCC [227]. The incidence of AF in association with other VEGFIs is limited to case reports and it can be difficult to confidently ascribe causation, which may be multifactorial in patients with cancer, to VEGFI [228,229]. Similar to the other ‘multiple hit’ hypotheses outlined, the development of VEGFI-associated AF may be potentiated by other myocardial toxic effects in which the effects of cardiomyocyte toxicity, LV contractile dysfunction or hypertension may create a substrate for arrhythmia. The mechanisms of AF in relation to VEGFI treatment are not well-understood. However, one potential mechanism for VEGFI-induced AF may be via the inhibition of the cardiomyocyte PI3k-Akt pathway. As outlined above, VEGF binding to VEGFR-2 on endothelial cells is associated with increased cell survival and migration via the PI3k-Akt pathway and inhibition of PI3k-Akt signalling has been implicated in the development of AF in mouse models [48,51,230]. Mice expressing a dominant-negative mutant of PI3k develop AF while, in that model, increasing PI3k activity reduced atrial fibrosis and improved conduction [230]. In keeping with this, atrial appendage PI3k activity is lower in patients with AF than it is in those with sinus rhythm [230].

## Conclusions

VEGFIs are associated with cardiovascular toxicities including myocardial dysfunction affecting both left and right ventricles, and conduction abnormalities leading to cardiac arrhythmias. These may occur via on-target effects on VEGF and its receptors or via off-target effects on other receptors. These effects may be exerted directly upon cardiomyocytes, or indirectly via the coronary and peripheral vasculature. Additionally, they may be exacerbated by previous or concurrent myocardial injury from the use of other cardiotoxic anti-cancer therapies including anthracyclines and ICIs. Ultimately however, LV dysfunction is likely to result from the combination of multiple insults on the myocardium rather than any individual mechanism alone.

Although the mechanisms underlying these cardiovascular toxicities are becoming better understood, substantial gaps in knowledge remain. The limited number of head-to-head studies, as well as inadequacies in reporting and description of adverse cardiac effects in trials, makes it difficult to make robust comparisons between cardiotoxicity profiles for each VEGFI drug or class. It is imperative that clinically focussed translational research continues so that patients can achieve large oncological clinical benefits from these drugs without potentially unacceptable cardiovascular side effects. Refining our mechanistic understanding of the cardiovascular toxic effects of VEGFIs will provide a stronger platform from which to test and implement cardiovascular preventive strategies and treatments. This will also inform best cardiovascular surveillance for patients receiving these potent drugs. Ultimately, well-designed clinical trials assessing cardiovascular toxicities in VEGFI-treated patients with cancer should provide information for much-needed evidence based clinical guidelines.

## Abbreviations

ACE: angiotensin-converting enzyme
AF: atrial fibrillation
AMPK: adenosine monophosphate kinase
AP: action potential
ASK-1: apoptosis signal-regulating kinase 1
ATP: adenosine triphosphate
AV: atrioventricular
Bcl-2: B cell lymphoma-2
CAD: coronary artery disease
CTEPH: chronic thromboembolic pulmonary hypertension
*ded*: *dead beat*
ECG: electrocardiogram
EDHF: endothelial-derived hyperpolarising factor
eNOS: endothelial NO synthase
ERK1/2: extracellular signal-regulated kinase
ESMO: European Society of Medical Oncology
ET-1: endothelin-1
FAK: focal adhesion kinase
FLT3: fms-like tyrosine kinase-3
HCC: hepatocellular carcinoma
hERG: human ether-a-go-go related gene
HF: heart failure
HFpEF: heart failure with preserved ejection fraction
HIF-α: hypoxia inducible factor-α
HUVEC: human umbilical vein endothelial cells
ICI: immune checkpoint inhibitor
IKr: rapid delayed rectifier current
LVEF: left ventricular ejection fraction
LVSD: left ventricular systolic dysfunction
MAPK: mitogen-activated protein kinase
MEK: MAPK/ERK kinase
MI: myocardial infarction
mRNA: messenger ribonucleic acid
miRNA: micro-ribonucleic acid
MST-2: mammalian Ste20-like kinase-2
mTOR: mechanistic target of rapamycin
NAC: N-acetyl-cysteine
NCI: National Cancer Institute
NF-B: nuclear factor kappa-light-chain-enhancer of activated B cells
NO: nitric oxide
PD-1: programmed cell death protein-1
PDGFR: platelet-derived growth factor receptor
PDK1: 3-phosphoinositide dependent protein kinase-1
PD-L1: programmed death-ligand-1
PGI_2_: prostacyclin
PH: pulmonary hypertension
PHD: prolyl hydroxylase domain
PI3K: phosphatidyl inositol-3 kinase
PLC-γ: phospholipase C
PKC: protein kinase C
QTc: corrected QT interval
RAA: renin angiotensin aldosterone
Raf-1: rapidly accelerated fibrosarcoma-1
RCC: renal cell cancer
R-CHOP: rituximab cyclophosphamide doxorubicin vincristine prednisolone
RET: rearranged during transfection
ROS: reactive oxygen species
RR: relative risk
RV: right ventricular
SCD: sudden cardiac death
SCF: stem cell growth factor
SPRED-1: sprout-related EVH1 domain-containing protein-1
TAC: transverse aortic constriction
TAPSE: tricuspid annular plane systolic excursion
TdP: Torsades de Pointes
TKI: tyrosine kinase inhibitor
VEGF: vascular endothelial growth factor
VEGFI: vascular endothelial growth factor inhibitor
VE-cadherin: vascular endothelial-cadherin
VF: ventricular fibrillation
VT: ventricular tachycardia
XIAP: X-linked inhibitor of apoptosis protein
